# Monocular central retinal artery occlusion caused by bilateral internal carotid artery hypoplasia complicated with patent foramen ovale: a case report and review of literature

**DOI:** 10.1186/s40001-021-00530-w

**Published:** 2021-06-13

**Authors:** Lanbing Zhu, Na Xu, Yan Li

**Affiliations:** Department of Neurology, The Second Affiliated Hospital and Yuying Children’s Hospital of Wenzhou Medical University, 109 Xueyuan Road, Wenzhou, 325027 Zhejiang China

**Keywords:** Case report, Central retinal artery occlusion, Internal carotid artery, Hypoplasia, Patent foramen ovale

## Abstract

**Background:**

Central retinal artery occlusion (CRAO) is an emergent ophthalmic disease which is commonly caused by atherosclerosis, thromboembolism, and arteriospasm. Here, we report a case of CRAO which is caused by extreme rare bilateral internal carotid artery (ICA) hypoplasia complicated with patent foramen ovale (PFO). The cardiogenic emboli blocked central retinal artery through unclosed foramen ovale and specific blood flow pathway.

**Case presentation:**

This report describes a case of a 46-year-old woman sudden onset with amaurosis fugax for about 20 min and persistent visual impairment of left eye. Fundus fluorescein angiography shows the arm-retinal circulation time of left eye is 25 s, indicating that the occlusion occurs in the pathway from aortic arch to ophthalmic artery. The MRA and CTA examinations reveal the bilateral ICA hypoplasia and variation of Wills circle. Furthermore, transesophageal echocardiography (TEE) confirms the PFO and cardiogenic embolic event.

**Conclusions:**

This work presents a CRAO case caused by rare congenital hypoplasia of ICA complicated with PFO, reminding us every single cause of vascular disease should be investigated carefully and the TOAST typing of cerebrovascular disease can be of great reference to the ocular vascular disease.

**Supplementary Information:**

The online version contains supplementary material available at 10.1186/s40001-021-00530-w.

## Background

As the internal carotid artery (ICA) is one of the most important blood supply arteries to the brain, the hypoplasia of ICA is rare [1] and the bilateral occurrence is even rarer [2]. Most patients with ICA hypoplasia are asymptomatic owing to the compensatory collateral communication of the intracranial arteries [3]. Central retinal artery occlusion (CRAO) is an emergent ophthalmic disease with sudden onset of blindness of the affected eye. The common reasons for the disease are atherosclerosis, thromboembolism, and arteriospasm. Here, we report a case of CRAO which is caused by extreme rare bilateral internal carotid artery (ICA) hypoplasia complicated with patent foramen ovale (PFO). The cardiogenic emboli blocked central retinal artery through unclosed foramen ovale and specific blood flow pathway.

## Case presentation

**Fig. 1:**
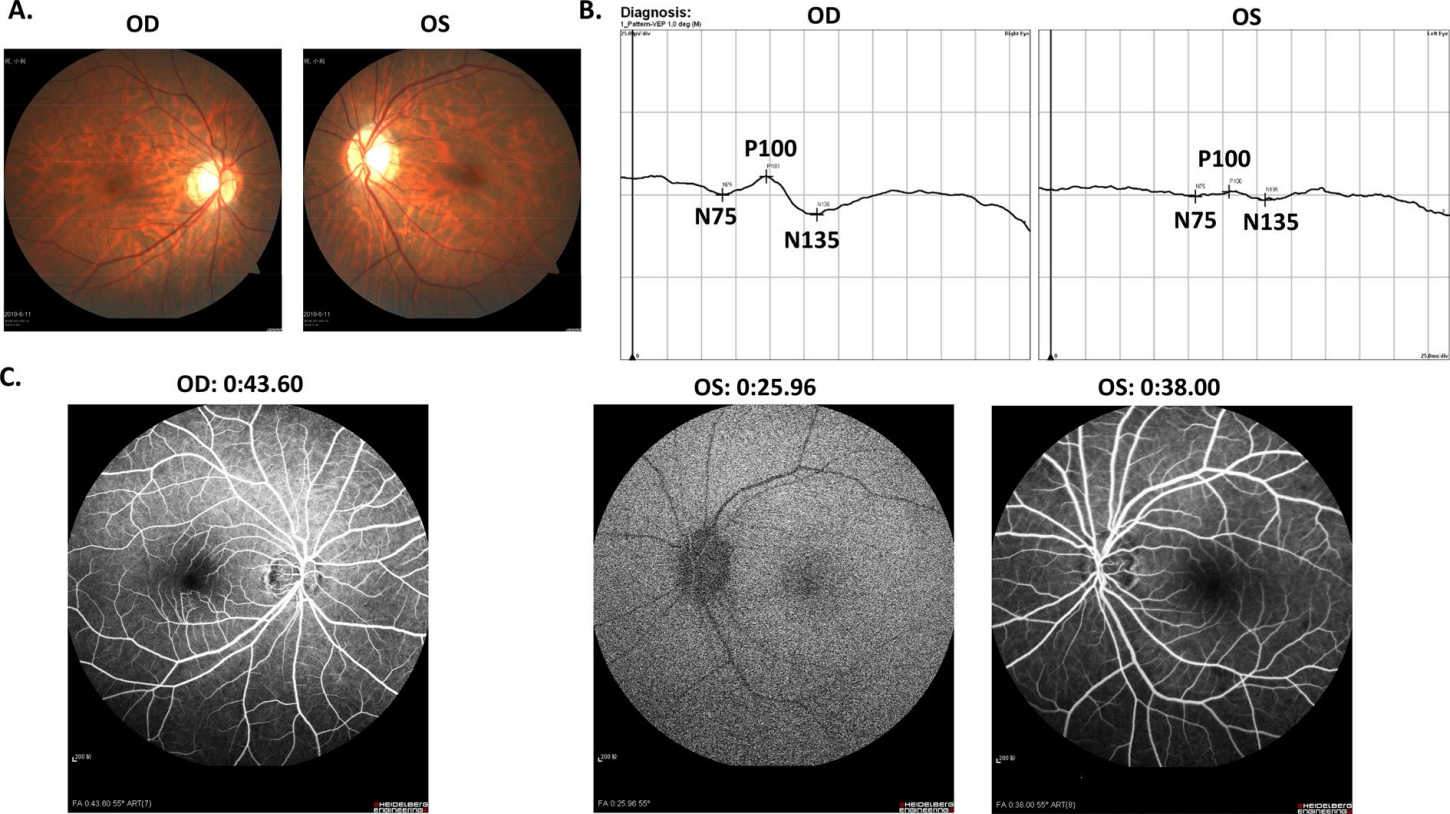
The ophthalmology examinations: **A** fundus color photography; **B** VEP test; **C** fundus fluorescein angiography with time counting from intravenous injection of contrast medium from cubital vein. *OD* right eye, *OS* left eye

A 46-year-old woman with no previous medical history first visited ophthalmologic department with amaurosis fugax for about 20 min and persistent visual impairment of left eye. The corrective vision examination showed that visual accuracy of the left eye is 0.1 and the right eye is normal 1.0. Pupillary light reflex test showed relative afferent pupillary defect (RAPD) of the left eye. The fundus examination did not find significant abnormalities (Fig. 1A). The Visual-Evoked Potential (VEP) test demonstrated the prolonged latency and declined amplitude of P100 wave of the left eye (Fig. 1B), indicating the impairment of left visual pathway. The fundus fluorescein angiography found that the arm-retinal circulation time of left eye was 25 s (Fig. 1C and Additional file 1: Video 1), whereas the normal time window is only 10 ~ 15 s. The right eye did not present any abnormality (Fig. 1C). Accordingly, the CRAO diagnosis was confirmed and ophthalmologic examination indicated that the occlusion might occur in the pathway from aortic arch to ophthalmic artery.

**Fig. 2:**
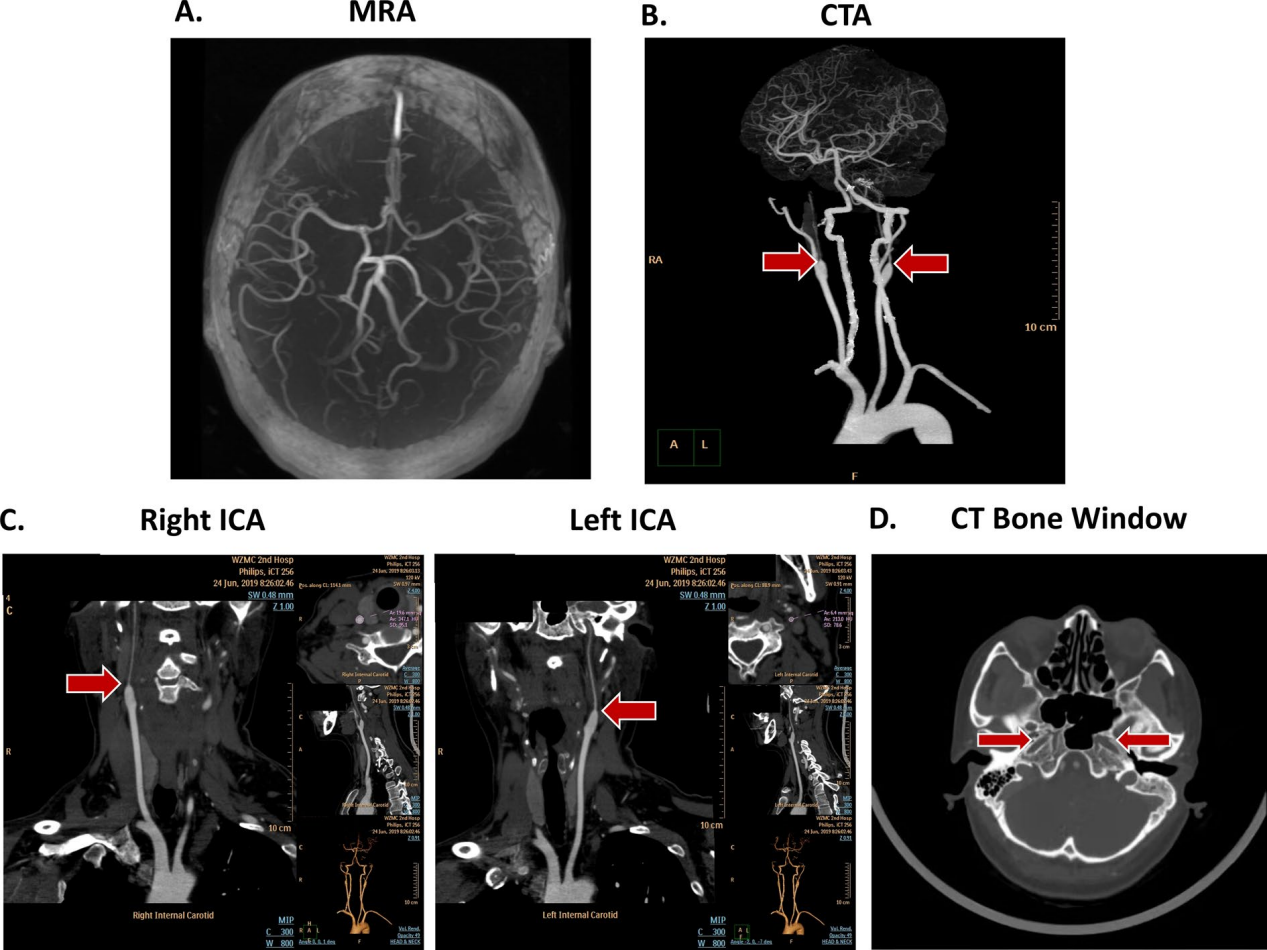
The neuroimaging examinations: **A** MRA; **B** CTA; **C** the re-established images of bilateral ICA. **D** CT bone window

Therefore, the patient was transferred to neurological department. The MR angiography (MRA) found that bilateral ICAs were absent concurrent with dilated vertebral and basilar arteries. Meanwhile, there were variations in the circle of Willis with opened right posterior communication artery (PCoA) and absence of left PCoA (Fig. 2A). Moreover, CT angiography (CTA) images (Fig. 2B, C) illustrated that bilateral hypoplasia of the ICAs from carotid bifurcation with thin remnants and the bilateral bony carotid canals still existed in CT bone window (Fig. 2D), confirming the diagnosis of bilateral ICAs hypoplasia but not ICAs agenesis. Hereto, we can depict the characteristics of the blood flow of the case: (1) due to bilateral ICAs hypoplasia, vertebrobasilar artery system takes the responsibility to provide blood supply to the whole brain and results in dilated vertebral and basilar arteries; (2) as a result of variation of the circle of Willis, the blood flow first supplies right middle cerebral artery (MCA) and ophthalmic artery via opened right PCoA and then the left MCA and ophthalmic artery via opened anterior communicating artery (ACoA).

**Fig. 3:**
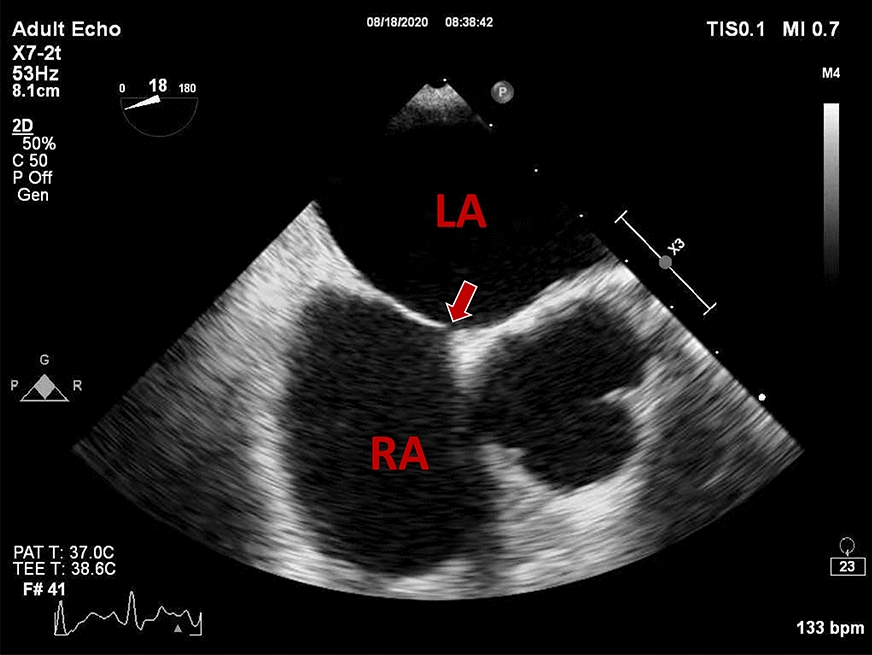
TEE image. *LA* left atrium, *RA* right atrium. The arrow points to the PFO

However, the amaurosis fugax and lasting visual impairment of left eye cannot be well interpreted. According to the standard of TOAST typing of stroke, the cardiogenic factors were evaluated then. Atrial fibrillation and valve vegetation were not detected by 24-h dynamic electrocardiogram and echocardiograms. Transesophageal echocardiography (TEE) revealed patent foramen ovale (Fig. 3) and blood split-flow from right to left atrium when doing Valsalva breath during the Foaming test (Additional file 2: Video 2). Therefore, the amaurosis fugax and persistent visual impairment of left eye was caused by cardiogenic embolism.

Aspirin 100 mg/day was given to the patient and PFO occlusion surgery was recommended by us. However, the patient refused the surgery and was lost to follow-up.

## Discussion and conclusions

Here, to the best of our knowledge, we first present a case of CRAO caused by bilateral ICA hypoplasia complicated with patent foramen ovale. The embolus migrates following the specific pathway: right atrium → unclosed foramen ovale → left atrium → aortic arch → dilated vertebrobasilar artery system → right PCoA → right anterior cerebral artery (ACA) → ACoA → left ACA → left ophthalmic artery/left central retinal artery. As the ICA is one of the most important blood supply arteries to the brain, the anatomical variation of ICA is rare. ICA hypoplasia was first reported by autopsy in 1787. The hypoplasia occurs in less than 0.01% of the population [1] and the bilateral occurrence is even rarer (only < 10% of those cases) [4]. Although the cause of ICA hypoplasia is still undefined, the abnormal ICA development is the widely accepted view [5]. Briefly, any insult to the ICA embryologic origin can result in abnormal development of ICA. The ICA completes development in the fourth week of embryo, whereas the bony carotid canal develops secondary to the presence of fetal ICA in the fifth-to-sixth week of embryo. Therefore, the absence of carotid canal signifies the complete developmental failure of ICA [6], the so-called agenesis. On the other hand, the terms of aplasia and hypoplasia refer to the defective development of ICA with existing bony carotid canal and remnant vessels in the canal, as our case shows. It should be noted that in the literature, agenesis, aplasia, and hypoplasia are often used interchangeably.

Anatomically, six types of collateral circulation in ICA hypoplasia cases were described by Lie et al. [3, 7]. Type A, unilateral absence of the ICA with compensatory blood flow to affected ACA from contralateral ACA via ACoA and to affected MCA via PCoA. Type B, ipsilateral ACA and MCA on the side of absent ICA are supplied by contralateral ACA via the ACoA. Type C, bilateral ICA hypoplasia with compensatory blood supplies to bilateral ACAs and MCAs via PCoAs. Type D, hypoplasia of the cervical segment of the ICA with the cavernous segment of the ICA reconstituted by an anastomotic siphon from the contralateral cavernous ICA. Type E, hypoplasia but not agenesis of bilateral ICA characterized by diminutive ICAs. The bony carotid canals persist in this type which can be used for differential diagnosis. Type F, the absence of proximal ICA but with distal ICA reconstitution by anastomosis with distal branches of external carotid artery (ECA). Additionally, the hypoplasia of ICA with persistent trigeminal artery was also reported [8]. Our case with the characteristics of both type B and type E cannot be classified as either of the six types.

Because of the compensatory effect of collateral circulation, most ICA hypoplasia cases are asymptomatic. Some cases may have headache, seizures, transient ischemic attack, and subarachnoid hemorrhage occasionally [9]. Additionally, some symptoms, such as trigeminal neuralgia, oculomotor paralysis, visual field defect, or vision loss, are caused by dilated artery (basilar artery and ACoA) or the aneurysm formed there [9–12]. Other rare syndromes, e.g., Hemangiomas, Posterior fossa brain malformations, CADASIL, Arterial lesions, Goldenhar syndrome, Cardiac abnormalities/aortic coarctation and Eye (PHACE) abnormalities, Klippel–Feil syndrome, coarctation of aorta, hypopituitarism, and growth hormone deficiency, have also been reported concurrent with ICA hypoplasia with no clear reasons [13–19].

The diagnosis of ICA hypoplasia mainly depends on angiography, MRA, CTA, and DSA [1]. The following characteristics can be taken for diagnosis [3]: (1) the absence of ICA and carotid canal (in the bony CT window), (2) the compensatory collateral circulation, e.g., dilated basilar artery, PCoA, ACoA, and variations of Willis circle, and (3) lack of risk factors of atherosclerosis for arterial occlusion, such as hypertension, diabetes, and hyperlipidemia. Given the congenital and asymptomatic natures of carotid hypoplasia in most cases, no treatment is necessary to re-establish the ICA. The therapeutic plans mainly depend on the diseases secondary to ICA hypoplasia, e.g., ruptured intracranial aneurysms.

This case reminds us every single cause of vascular disease should be investigated carefully, especially for some uncommon clinical presentations and the TOAST typing of cerebrovascular disease can be of great reference to the ocular vascular disease.

## Supplementary Information

**Additional file 1.** The fundus fluorescein angiography of left eye.

**Additional file 2.** The Foaming test of Transesophageal echocardiography (TEE).

## Data Availability

The data generated during the present study are available upon request from the corresponding author.

## Abbreviations

CRAO: Central retinal artery occlusion
ICA: Internal carotid artery
PFO: Patent foramen ovale
RAPD: Relative afferent pupillary defect
VEP: Visual-Evoked Potential
PCoA: Posterior communication artery
MCA: Middle cerebral artery
ACoA: Anterior communicating artery
ACA: Anterior cerebral artery
ECA: External carotid artery
